# Integrated Nutritional, Spectroscopic and Technological Evaluation of Black Oat (*Avena strigosa*) and White Oat (*Avena sativa* L.) Cultivars

**DOI:** 10.3390/molecules31040639

**Published:** 2026-02-12

**Authors:** Bogdan Cozma, Sylvestre Dossa, Antoanela Cozma, Daniela Stoin, Dacian Lalescu, Isidora Radulov, Ilinca Imbrea, Georgeta Pop, Laura Crista, Mariana Suba, Ersilia Alexa, Florin Imbrea

**Affiliations:** 1 Faculty of Agriculture, University of Life Sciences “King Mihai I” from Timisoara, Aradului Street No. 119, 300645 Timisoara, Romania; cozmabogdan2014@gmail.com (B.C.); isidora_radulov@usvt.ro (I.R.); ilinca_imbrea@usvt.ro (I.I.); georgeta_pop@usvt.ro (G.P.); laura.crista@usvt.ro (L.C.); florin_imbrea@usvt.ro (F.I.); 2 Doctoral School, University of Life Sciences “King Mihai I” from Timisoara, Aradului Street No. 119, 300645 Timisoara, Romania; ersiliaalexa@usvt.ro; 3 Faculty of Food Engineering, University of Life Sciences “King Mihai I” from Timisoara, Aradului Street No. 119, 300645 Timisoara, Romania; dossasylvestre@usvt.ro (S.D.); danielastoin@usvt.ro (D.S.); lalescu@usvt.ro (D.L.); 4 “Food Science” Research Center, University of Life Sciences “King Mihai I” from Timisoara, Aradului Street No. 119, 300645 Timisoara, Romania; 5 Romanian Academy, “Coriolan Dragulescu” Institute of Chemistry, Mihai Viteazu No. 24, 300223 Timișoara, Romania; marianasuba@gmail.com

**Keywords:** bread, proteins, lipids, total mineral contents, polyphenols, MIXOLAB, FTIR

## Abstract

Oat is increasingly recognized as a valuable cereal due to its favorable nutritional profile and potential application in functional foods. This study aimed to provide an integrated nutritional and technological evaluation of black oat (*Avena strigosa*) and white oat (*Avena sativa* L.) cultivars Ovidiu, Jeremy, and Sorin, grown under uniform conditions. The chemical composition was assessed by determining proteins, lipids, total mineral and polyphenol contents. Macro- and microelement profiles (Ca, Mg, K, Na, Fe, Mn, Cu, Ni, and Zn) were quantified by atomic absorption spectrometry (AAS), while the technological suitability of black oat flour for bakery applications was evaluated using Mixolab analysis and bread quality parameters. Additionally, Fourier-transform infrared (FTIR) spectroscopy was applied to investigate structural features associated with β-glucans in the oat samples. The results showed that protein content ranged from 12.39 to 13.48%, while lipid content varied between 3.24 and 4.64%. Significant differences were observed in mineral composition among the analyzed samples. Black oat showed a balanced mineral profile, characterized by high levels of K, Mg, Mn, Zn, and Ni, confirming its classification as a mineral-rich cereal, while the Ovidiu cultivar generally presented the lowest concentrations for most elements. Mixolab results revealed that the partial substitution of wheat flour with black oat flour significantly influenced dough rheological behavior, particularly in terms of protein weakening and starch gelatinization, without severely affecting dough stability when applied at moderate inclusion levels. Bread quality evaluation demonstrated acceptable crumb elasticity, porosity, and height-to-diameter ratios, supporting the feasibility of incorporating black oat in bakery products. FTIR analysis revealed characteristic absorption bands associated with β-glucans, supporting their presence and structural integrity in both black oat and cultivated varieties. Overall, this study demonstrates that both black oat and selected oat cultivars represent valuable raw materials for functional food applications, offering enhanced nutritional profiles and suitable technological performance. The combined use of compositional, rheological, and spectroscopic analyses provides a comprehensive approach for evaluating oat-based ingredients in the context of modern cereal science.

## 1. Introduction

Oat (*Avena* spp.) is a cereal of increasing scientific and industrial interest due to its exceptional nutritional profile and its potential application in functional food products. Compared to other cereals, oats are distinguished by a higher content of soluble dietary fiber, high-quality proteins, unsaturated lipids, and bioactive compounds, all of which contribute to various beneficial health effects, including modulation of lipid metabolism, glycemic control, and cardiovascular protection [1,2,3]. Recent research on oats also highlights their beneficial role for health: soluble fibers and β-glucans contribute to regulating blood lipids, maintaining cardiovascular health, and controlling blood glucose; unsaturated fats and micronutrients (minerals, B vitamins, and antioxidant vitamins) provide a valuable nutritional profile [4,5]. The most widely cultivated species for human consumption is *Avena sativa* L., which is characterized by stable yields and suitability for food processing. In contrast, *Avena strigosa*, commonly known as black oat, has traditionally been cultivated mainly for forage purposes but has recently gained attention due to its resilience to abiotic stress, higher mineral content, and elevated levels of phenolic compounds [6,7]. These characteristics make black oat a promising raw material for both nutritional and technological applications.

From a nutritional perspective, oat grains contain between 12 and 16% protein, exceeding the protein content of wheat and maize. Oat proteins are primarily composed of globulins and avenins, which exhibit a more balanced amino acid profile, particularly with respect to lysine [1]. Several studies report that *Avena strigosa* may exhibit slightly higher crude protein and dietary fiber contents compared to *Avena sativa*, suggesting potential advantages for the development of low-glycemic or high-fiber food products [7,8]. For black oats, Brindzová et al. (2008) reported a relatively high content of phenolic compounds and antioxidant activity compared to light-coated varieties (yellow/naked) [8].

Due to this composition, black oats show significant potential for use in balanced diets, as well as in vegetarian, vegan, or health-oriented diets. A distinctive aspect of black oats is their high content of β-glucans and phenolic compounds, whose beneficial effects on health are well documented. These bioactive constituents may contribute to reducing cardiovascular risk, regulating the postprandial glycemic response and combating oxidative stress, thus supporting the inclusion of black oats in preventive nutritional strategies [7].

Regarding micronutrient composition, oats represent an important dietary source of essential minerals, including magnesium (Mg), phosphorus (P), iron (Fe), zinc (Zn), and manganese (Mn), as well as B-complex vitamins. Comparative studies indicate that black oat grains may contain higher concentrations of certain microelements, particularly Fe and Mn, which are critical for enzymatic processes and oxidative stress regulation [4,5,6].

From a technological standpoint, the use of oat flour in bakery products is limited by the absence of gluten networks comparable to those in wheat-based systems. Nevertheless, incorporating oat flour improves water-holding capacity, nutritional density, and the shelf life of baked goods [9]. Black oat flour, in particular, may affect crumb structure, color, and texture due to its higher fiber content and darker pigments, offering opportunities for the development of novel cereal-based products with enhanced functional properties [10].

Previous studies indicate that oat genotype and kernel pigmentation can significantly influence the level of bioactive compounds and technological performance [8,11,12]. In particular, colored/black oats have often been associated with higher total phenolic content and antioxidant capacity compared with common (white/yellow) oats, with pigments such as anthocyanins contributing to these differences. At the same time, research has highlighted substantial variability among oat cultivars in key functional components such as β-glucan and lipids, supporting the importance of cultivar-level evaluation rather than treating oats as a uniform raw material [13,14]. These findings justify comparative studies that connect varietal biochemical profiles to processing behavior and final product quality in cereal-based foods [14].

The reduction or partial replacement of conventional flour with black oat flour in bakery products offers the opportunity to develop functional foods with improved nutritional value. Although the absence of gluten limits the formation of the structural network specific to wheat dough, the use of black oats in moderate proportions allows for obtaining products with acceptable sensory and structural properties, while increasing the content of fiber, protein and antioxidants.

Given the growing demand for sustainable and health-oriented foods and for valorization of alternative cereals, a comparative evaluation of common oat (*Avena sativa* L.) and black oat (*Avena strigosa*) in terms of macronutrients, microelements, and baking-related properties is highly relevant. Such analyses provide a scientific basis for expanding the use of alternative oat species in human nutrition and food processing.

In recent years, numerous studies have investigated the nutritional and functional properties of oats, as well as their incorporation into wheat-based products. However, most research refers to generic oat types or internationally widespread cultivars, while limited information is available regarding varieties cultivated in Eastern Europe.

The present study focuses on the oat varieties Ovidiu, Jeremy, and Sorin, which are Romanian cultivars widely adapted to local pedoclimatic conditions and grown at an agricultural scale. Despite their agronomic relevance, data concerning their technological performance in composite wheat–oat systems and their influence on bread quality and functional characteristics remain scarce. Evaluating these varieties is therefore important not only from a scientific perspective, but also for their practical applicability in regional cereal processing. By linking varietal composition with dough rheology, bread structure, and bioactive potential, this work contributes to a better understanding of the suitability of locally cultivated oats for bakery applications.

To our knowledge, no previous study has provided an integrated evaluation of compositional, rheological, technological, and functional characteristics specifically for these Romanian oat varieties relative to breadmaking performance. Therefore, the choice of these varieties was not arbitrary but based on their regional agronomic relevance and the need to generate data applicable to local cereal production and processing. Evaluating their performance in breadmaking provides useful information for both breeders and the baking industry regarding the technological suitability and functional potential of locally cultivated oats.

The novel and innovative elements of the present study consist in the implementation of an integrated analysis which combines the evaluation of the detailed nutritional composition (proteins, lipids, total mineral substances, polyphenols, and macro- and microelement profile) with the structural FTIR analysis, the rheological behavior of the dough and the quality of the bakery products obtained by adding black or white oat cultivars and assessing bread physical parameters. Specifically, this study combines molecular characterization (FTIR), advanced dough rheology (Mixolab), and detailed bread quality parameters (crumb elasticity, porosity, and color), together with the determination of polyphenols, antioxidant activity, and mineral composition. Such a comprehensive approach linking oat type → dough behavior → bread structure → functional properties has not been previously reported for the direct comparison between black and white oats.

In the absence of standardized data reported in the specialized literature that differentiate the nutritional values at the cultivar level for white oats (Ovidiu, Jeremy, and Sorin) and black oat, this study provides original experimental data that allows an objective comparison between oat varieties. In particular, the use of the Mixolab method to correlate protein properties, starch gelatinization and gel stability in dough systems enriched with black oats, as well as the concurrent evaluation of bread quality parameters (crumb elasticity, porosity and H/D ratio), represents an innovative approach to characterizing the technological potential of black oats in bakery production. The results obtained contribute to expanding the knowledge base on the valorization of alternative cereals and support the development of functional bakery products with improved nutritional profiles and practical applicability in the food industry. Additionally, correlating FTIR results with nutritional and technological data contributes to a deeper understanding of the relationship between chemical composition and the technological behavior of oats in baking applications, a topic insufficiently explored in recent specialized literature.

## 2. Results and Discussion

### 2.1. Nutritional Composition of Black Oat (Avena strigosa) and White Oat (Avena sativa *L.*) Cultivars

The protein and lipid contents of the analyzed samples fall within the ranges reported in the literature for oats (*Avena sativa* and *Avena strigosa*), confirming the high nutritional value of this cereal. The protein values obtained vary between 12.39% (black oat) and 13.48% (Ovidiu cultivar), with intermediate levels for Jeremy (12.48%) and Sorin (13.13%). This range is consistent with recently published data, which indicate that for oats the protein content generally ranges between 10.0 and 15.5%, depending on the genotype, cultivation conditions and nitrogen fertilization level [1,4,6]. The differences observed between varieties are relatively moderate, which is in agreement with the literature, emphasizing that although the genotype significantly influences the protein content, intra-specific variations are often limited when the crops are grown under similar conditions [5,6].

The proximate composition of black oat (*Avena strigosa*) and White Oat (*Avena sativa* L.) cultivars is presented in Table 1.

In terms of lipid fraction, the determined values show a more pronounced variability, ranging between 3.24% for the Ovidiu cultivar and 4.64% for the Sorin cultivar. Black oats (4.09%) and Jeremy (3.27%) are located between these extremes. The specialized literature shows that oats are distinguished from other cereals by a relatively high lipid content, usually between 3.0 and 7.0%, dominated by unsaturated fatty acids [1,4]. The higher values observed in Sorin and black oats are comparable to those reported for genotypes selected or cultivated under conditions favorable to lipid accumulation, suggesting a superior energetic and functional potential.

Compared to published data on black oats, the literature frequently indicates similar or slightly lower protein levels compared to white oats, but an equal or even higher lipid content, an aspect also confirmed by the results of the present study [9]. Furthermore, differences between varieties in lipid content are considered in the recent literature to be more sensitive to genetic variation than those observed for protein, which explains the larger range of lipid values obtained [4,5].

Overall, the results confirm that all the variants analyzed fit the profile characteristic of oats as an important plant source of high-quality proteins and lipids. The Ovidiu and Sorin varieties are distinguished by a slightly higher protein content, while Sorin and black oats present the highest lipid levels, supporting their potential for use in food products with improved nutritional and energy value. At the same time, the observed variations emphasize the determining role of genotype, in interaction with agroecological factors, on the chemical composition of oat grains.

The total mineral content revealed significant differences between the types of oats analyzed, confirming the pronounced influence of the genetic factor on the accumulation of mineral elements in the grain. The values obtained ranged between 1.52% and 2.47%, with the highest levels recorded for black oat (BO) and the Sorin (SO) variety, which presented statistically similar values (2.47 ± 0.36% and 2.26 ± 0.46%, respectively). In contrast, the Ovidiu (OO) and Jeremy (JO) varieties recorded significantly lower values (1.52–1.63%), indicating a lower mineral potential compared to BO and SO. The high level of mineral substances observed in black oats is consistent with the literature, which reports that pigmented or less improved forms of oats tend to accumulate higher amounts of macro- and microelements, including K, Mg, Ca, Fe, Zn, and Mn. This behavior is often associated with a less refined grain structure and a higher proportion of shell components, where minerals are concentrated. Recent studies confirm that black oats have a higher mineral density compared to conventional white oats, making them suitable as a functional ingredient for food fortification [3,6,9].

Similarly, the Sorin variety demonstrated a high content of mineral substances, close to that of black oats. This result suggests that, although Sorin is a modern cultivated variety, it retains favorable mineral accumulation characteristics, possibly due to genetic peculiarities or adaptation to specific pedoclimatic conditions. The literature indicates that certain modern oat varieties can achieve high mineral levels when bred for stress tolerance and nutritional efficiency, without completely compromising technological quality.

The lower values of mineral substances observed for Ovidiu and Jeremy are characteristic of conventional white oats and may be associated with a higher proportion of endosperm in relation to the outer fractions of the grain, where minerals are predominantly located. However, these varieties may offer complementary technological and nutritional advantages (e.g., higher protein or lipid content), which highlights the need for an integrated assessment when selecting raw materials for food applications.

From a nutritional point of view, differences in mineral content are particularly relevant, as minerals play an essential role in energy metabolism, enzymatic function, and the maintenance of bone health. Therefore, the use of black or Sorin oat varieties in food formulations, including bakery products, can significantly contribute to increasing the mineral density of the finished product. These results support the conclusions of other studies that highlight oats as a cereal with important potential for the development of functional foods and for diversifying dietary sources of minerals.

The carbohydrate content varied significantly among the oat samples analyzed. The highest value was recorded for JO (Jeremy) (73.10 g/100 g), followed by OO (Ovidiu) and BO (black oat), which did not differ significantly from each other. In contrast, the cultivar SO (Sorin) presented a significantly lower carbohydrate content (67.36 g/100 g).

These differences reflect the compositional variability specific to oat genotypes, frequently reported in the literature. Since carbohydrates were calculated by difference, the values are indirectly influenced by the levels of protein, lipids and ash. The lower carbohydrate content in Sorin may be associated with a higher proportion of other components (e.g., proteins, lipids or mineral fractions), indicating distinct biochemical particularities of this cultivar.

The variability between cultivars is consistent with studies showing that genetic factors influence the distribution of macronutrients in oat grains, potentially affecting their technological behavior and nutritional value.

### 2.2. Macro- and Microelemental Profile of Black Oat (Avena strigosa) and White Oat (Avena sativa *L.*) Cultivars

All oat samples are characterized by a rich mineral profile, with marked differences between black oats and the cultivated varieties Ovidiu, Jeremy, and Sorin (Table 2).

Potassium is, as reported in the literature, the dominant macroelement in oat grains, followed by magnesium and calcium, while sodium remains at low levels [15].

Interpretation of obtained data shows clear differences between black oats and the three white varieties (Ovidiu, Jeremy, Sorin), within the ranges recently reported for oats, but with some peculiarities. The contents of the macroelements Ca and K are highest in Sorin (Ca 511.60 mg/kg, K 2091.83 mg/kg), followed by Jeremy, while Ovidiu has noticeably lower values (Ca 234.39 mg/kg, Mg 247.82 mg/kg, K 655.37 mg/kg). Black oats has high K (1902.59 mg/kg) and Mg (655.14 mg/kg) content, and is still 1.8–2.9 times richer in Ca, Mg, and K than Ovidiu. These levels are within the ranges reported for various oat genotypes; for example, studies summarized by Alemayehu et al. (2023) and by Jakobsone et al. (2019) indicate for hulled oats values of the order of 420–835 mg/kg for Ca, 495–2000 mg/kg for Mg and approximately 2146–5621 mg/kg for K, depending on the genotype and agro-climatic conditions [5,15]. The obtained values for Mg (247.82–655.14 mg/kg) and Ca (234.39–511.60 mg/kg) are within or slightly below the ranges reported, confirming that the analyzed varieties have a good mineral potential, even if for K the concentrations are somewhat lower than those observed in some heavily fertilized genotypes or in very rich soil conditions [5,15].

Regarding microelements, all samples fall within the ranges described in the literature of the last decade: Fe—22.7–27.5 mg/kg, Mn—21.8–27.9 mg/kg, Cu—2.7–3.7 mg/kg, Ni—1.46–2.74 mg/kg and Zn—13.2–17.4 mg/kg. For Fe and Mn, the values are slightly below those reported by Jakobsone et al. for Latvian varieties (Fe between 33.5 and 48.9 mg/kg; Mn between 30 and 49 mg/kg) [15], but remain within the wide ranges synthesized by Alemayehu et al. (2023), who mention 3.0–43.9 mg/100 g for Fe and 2.6–19 mg/100 g for Mn (approximately 30–439 and 26–190 mg/kg, respectively) [5]. Zn content is moderate (13–17 mg/kg), slightly below the values of 22–32 mg/kg reported in studies of European genotypes and Zn biofortification experiments [15,16,17,18], but comparable to the ranges for “regular” oats in composition tables (16–34 mg/kg) [19]. Interestingly, black and Sorin oats have the highest levels of Zn and Cu, and black oats also have the highest Ni concentration 2.741 mg/kg), which is in agreement with recent observations that genotypic variability and agronomic management (fertilization, cropping system, soil type) can significantly modify the macro- and microelement profile of oats [15,16,17,18].

Overall, the results obtained highlight clear differences between the analyzed oat varieties in terms of mineral composition. The Sorin cultivar stood out for its highest concentrations of calcium, magnesium and potassium, associated with a high level of zinc, suggesting a superior nutritional potential among the genotypes investigated. Black oats presented a balanced mineral profile, characterized by high levels of potassium, magnesium, manganese and zinc, along with a relatively high concentration of nickel, confirming the conclusions of recent literature describing it as a valuable raw material in terms of macro- and microelement content [9].

The Jeremy cultivar occupied an intermediate position, with values close to those of black oats for most of the analyzed macro- and microelements, while the Ovidiu cultivar generally recorded the lowest concentrations for most of the determined elements. These results support the hypothesis that genetic variability between varieties is a determining factor in the mineral composition of oats, a variability that acts in close interaction with agroecological factors and crop management practices. Overall, the data obtained contribute to the consolidation of existing knowledge on the nutritional potential of oats and highlight opportunities for the exploitation of varieties with superior mineral profiles in human nutrition and in the development of functional food products.

**Figure 1:**
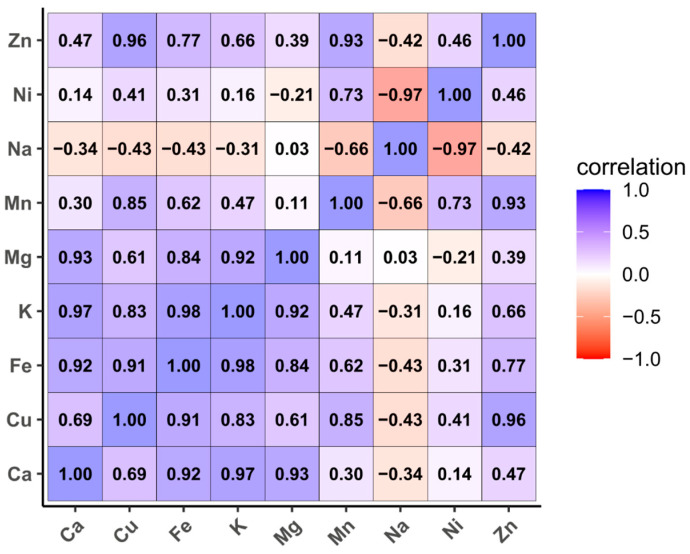
The Pearson’s product-moment correlation between macro and microelements.

Pearson’s correlation analysis revealed several strong and statistically significant relationships among the mineral elements (Figure 1). Calcium showed a very strong positive correlation with potassium (r = 0.97, *p* = 0.031), while potassium was also strongly and positively correlated with iron (r = 0.98, *p* = 0.017). In addition, a very strong positive association was observed between copper and zinc (r = 0.96, *p* = 0.036). In contrast, nickel exhibited a very strong and significant negative correlation with sodium (r = −0.97, *p* = 0.030).

Understanding the quantitative relationships among macro- and microelements is essential for elucidating mineral accumulation patterns and their nutritional relevance in cereal grains.

**Figure 2:**
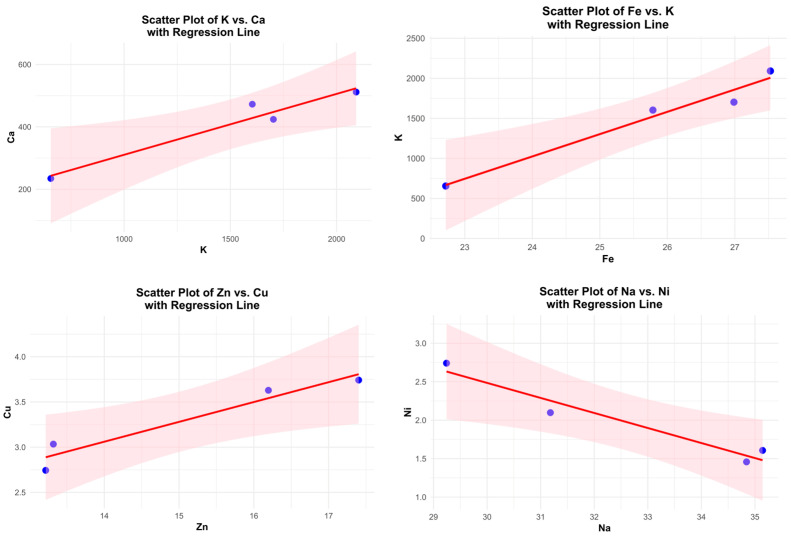
Linear regression analysis depicting the relationships between selected mineral elements: K vs. Ca, Fe vs. K, Zn vs. Cu, and Na vs. Ni. The solid red line represents the fitted linear regression model, while the blue dots correspond to the individual observed data points. The light red shaded area indicates the 95% confidence interval for the regression line.

Exploratory regression analysis has been proposed as a complementary approach to correlation analysis in order to better describe quantitative relationships among mineral elements. Previous studies have shown that strong inter-element correlations may reflect coordinated uptake, transport, or storage mechanisms within the plant matrix; however, such relationships are rarely explored using regression-based approaches, particularly for oat genotypes [20,21]. In this context, simple linear regression analysis provides a useful exploratory tool to describe the extent to which selected elements covary in a predictable manner. Simple linear regression analysis revealed strong and statistically significant linear relationships among selected macro- and microelements (Figure 2).

Potassium was a significant predictor of calcium content, explaining 93.9% of the variance (R^2^ = 0.94, *p* = 0.031), with calcium increasing proportionally with potassium concentration. In addition, iron showed a very strong positive linear association with potassium, accounting for 96.6% of the observed variability (R^2^ = 0.97, *p* = 0.017). At the micronutrient level, zinc significantly predicted copper concentration (R^2^ = 0.93, *p* = 0.036), indicating a closely coupled variation between these elements. Conversely, sodium exhibited a strong and significant negative linear relationship with nickel, explaining 94.2% of the variance (R^2^ = 0.94, *p* = 0.030), with increasing sodium concentrations associated with decreasing nickel levels. For all regression models, the estimated slopes were statistically significant, and the 95% confidence intervals confirmed the robustness of these linear trends.

**Figure 3:**
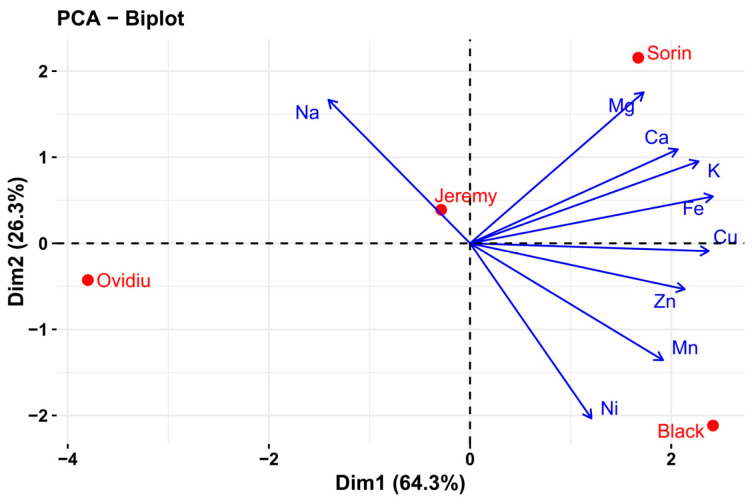
Biplot representation of macro- and microelement distribution in oat genotypes based on PCA.

The PCA biplot revealed a clear separation of oat genotypes based on their mineral composition, with the first two principal components explaining 90.6% of the total variance. Dim1 was mainly associated with K, Ca, Mg, Fe, Cu, Zn, Mn, and Ni, while Dim2 was driven primarily by Na. Strong positive associations were observed among K, Ca, Mg, and Fe, whereas Na showed an opposite trend relative to Ni. The genotypes Sorin and black were closely associated with higher mineral contents, whereas Ovidiu and Jeremy exhibited distinct and intermediate profiles, respectively (Figure 3).

### 2.3. Total Polyphenol Content and Antioxidant Activity (DPPH Assay) of Black Oat (Avena strigosa) and White Oat (Avena sativa *L.*) Cultivars

The total polyphenol content and antioxidant activity determined by the DPPH method revealed significant differences between the types of oat flour analyzed, confirming the strong influence of genetic origin on the bioactive potential of oats (Table 3).

The values obtained indicate that black oat flour (BO) presented the highest polyphenol content (408.59 ± 2.08 mg GAE/kg) and high antioxidant activity (54.68 ± 0.04%), highlighting its superior potential as a functional ingredient.

These results are consistent with data from the specialized literature, which reports that black oats and other pigmented forms of oats are richer in phenolic compounds, including avenanthramides, phenolic acids and flavonoids, compared to conventional white oat [22]. These compounds are recognized for their ability to neutralize free radicals and contribute to significant antioxidant and anti-inflammatory effects. Recent studies have shown that high levels of polyphenols in oats are directly correlated with antioxidant activity measured by radical methods, such as DPPH and ABTS [9].

Sorin oat flour (SO) showed the highest DPPH antioxidant activity (56.19 ± 0.07%), despite a lower polyphenol content (209.89 ± 0.33 mg GAE/kg). This observation suggests that antioxidant activity does not depend exclusively on the total amount of polyphenols, but also on the chemical structure and bioavailability of phenolic compounds, as well as the presence of other natural antioxidants, such as tocopherols and phytosterols. The literature indicates that certain avenanthramides and low molecular weight phenolic fractions can have high antioxidant efficiency even at lower concentrations [23].

In the case of Jeremy (JO) and Ovidiu (OO) oat flours, the values of polyphenol content (314.32 ± 0.00 and 260.09 ± 0.67 mg GAE/kg) and antioxidant activity (48.22 ± 0.07% and 49.95 ± 0.13%) were intermediate, indicating a moderate antioxidant potential. The differences observed between these varieties can be explained by genetic variations in the biosynthesis of phenolic compounds and by the distribution of different antioxidant classes. Previous studies have shown that modern oat varieties can present distinct phenolic profiles, even when grown under similar conditions, which supports the hypothesis of a significant genetic control of polyphenol content [24].

Overall, the results confirm that black oat is distinguished by a high content of polyphenols, while the Sorin variety presents the highest antioxidant activity, suggesting different antioxidant mechanisms between genotypes. This differentiation is particularly relevant for the development of functional bakery products, as it allows for selection of the type of oat depending on the nutritional objective pursued: enrichment in polyphenols (black oat) or maximization of antioxidant capacity (Sorin). The results are consistent with recent literature, which highlights the importance of oats as a valuable source of bioactive compounds with potential health benefits [24].

### 2.4. ATR–FTIR Spectroscopy of Black Oat (Avena strigosa) and White Oat (Avena sativa *L.*) Cultivars

FTIR was used as a supporting technique to identify characteristic functional groups and to highlight relative spectral differences among oat varieties within the context of flour systems.

**Figure 4:**
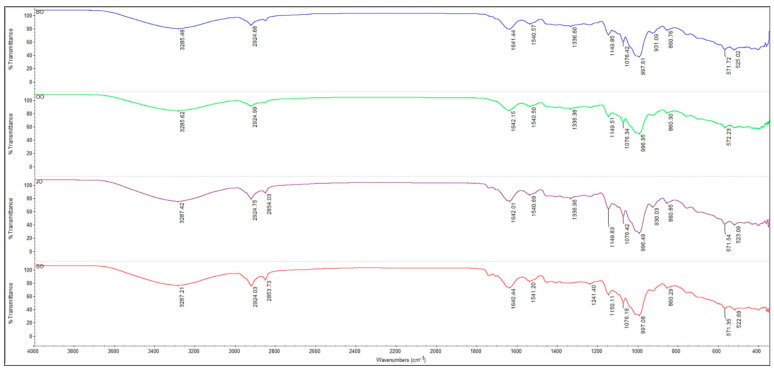
FTIR spectra of the oat samples (BO, JO, SO, and OO), spectral range of 4000–500 cm^−1^, 32 scans at 4 cm^−1^ resolution. BO: Black oat; OO: Ovidiu oat; JO: Jeremy oat; SO: Sorin oat. Line colors: BO—blue, OO—green, JO—mauve, SO—red.

The FTIR spectra of the analyzed oat samples (BO, OO, JO, and SO), recorded in the 4000–500 cm^−1^ range, exhibit a spectral fingerprint typical of a cereal matrix, dominated by contributions from polysaccharides (starch and β-glucan/hemicellulose fractions), with additional contributions from proteins and lipids (Figure 4).

The similar spectral profiles observed for all samples indicate a comparable underlying chemical matrix, largely governed by polysaccharides, in agreement with previous reports on cereals and, in particular, oat-based products analyzed by ATR–FTIR spectroscopy [25,26,27]. A broad and intense band is observed in the 3600–3000 cm^−1^ region, with maxima around ~3287–3285 cm^−1^, which can be attributed to O–H stretching vibrations. This band reflects the presence of abundant hydroxyl groups from polysaccharides, as well as hydrogen-bonded water, a characteristic feature of carbohydrate-rich food matrices. Similar observations have been reported for extracted and standard β-glucans, which exhibit FTIR absorption bands in the 3000–3500 cm^−1^ range assigned to hydroxyl groups within the polysaccharide structure, thereby confirming the dominant carbohydrate-based nature of these materials [28]. In the 3000–2800 cm^−1^ region, all spectra exhibit absorption bands at approximately 2924 cm^−1^ and 2853–2854 cm^−1^, corresponding to C–H stretching vibrations of aliphatic –CH_2_ and –CH_3_ groups, primarily associated with the lipid fraction. Such bands are commonly reported in ATR–FTIR spectra of cereal-based food matrices and their derivatives, where the intensity of the C–H stretching bands correlates with lipid content. The relatively similar intensities of these bands across the four samples indicate that the observed spectral differences are not predominantly driven by variations in lipid content, but rather by other components of the food matrix, as previously reported for cereals and plant-based materials analyzed by FTIR spectroscopy [29,30]. The 1700–1500 cm^−1^ region is dominated by absorption bands characteristic of proteins. The band observed in the ~1642–1600 cm^−1^ range can be assigned to the Amide I band, associated with C=O stretching vibrations of peptide bonds, with possible overlapping contributions from bound water within the cereal matrix. The band at ~1541–1540 cm^−1^ corresponds to the Amide II band, attributed to N–H bending and C–N stretching vibrations. The presence of these bands confirms the existence of a protein fraction in all analyzed samples, while variations in band position and intensity may reflect differences in the structural organization of proteins within the oat matrix. Similar interpretations of the amide region in FTIR/ATR–FTIR spectra of cereals and oats have been previously reported in the literature [25,27,30]. By comparison, sample BO exhibits a slightly more pronounced band definition in the amide region, suggesting a relatively higher contribution of the protein fraction and/or a different structural organization of proteins within the matrix. In the 1350–1200 cm^−1^ region, mixed contributions from carbohydrates and proteins are observed, including the Amide III region. Sample SO is distinguished by a relatively more intense band around ~1241 cm^−1^, a region associated in the literature with Amide III vibrations (C–N and N–H) and/or overlapping contributions from polysaccharides, indicating a different distribution of components or a distinct molecular environment in this sample [27]. The 1200–900 cm^−1^ region, known as the carbohydrate “fingerprint” region, exhibits the most intense bands across all analyzed samples and is associated with C–O–C and C–O stretching vibrations characteristic of starch and polysaccharides such as β-glucans and hemicelluloses. All spectra exhibit characteristic absorptions at approximately 1150–1149 cm^−1^, 1076 cm^−1^, and 997–996 cm^−1^, as well as, for some samples, around ~931 cm^−1^, which are attributed to glycosidic bond vibrations and skeletal vibrations of polysaccharides. This region is widely used for the evaluation of structural differences and molecular organization of carbohydrates in cereals and complex food matrices and has been extensively discussed in the literature in the context of spectroscopic characterization of starch and glucans [27,28,29]. Compared with the other samples, sample BO exhibits more pronounced bands in the carbohydrate region (1200–900 cm^−1^), as well as a more intense O–H profile, suggesting a relatively higher contribution of hydrophilic and polysaccharide components, given that all samples were analyzed under identical experimental conditions.

Considering that β-glucans represent a major functional constituent of oats, these spectral differences are consistent with reports linking higher polysaccharide contributions—particularly soluble β-glucans—to more favorable functional attributes, as described in the literature [26,30]. In the sub-900 cm^−1^ region, all oat varieties exhibit absorption bands or spectral shoulders in the 900–850 cm^−1^ range, as well as absorptions at approximately ~572–571 cm^−1^ and ~525–522 cm^−1^, which are attributed to skeletal vibrations and out-of-plane deformation modes of polysaccharide structures.

The 900–850 cm^−1^ region includes a marker frequently reported in the FTIR literature for β-(1→3), (1→4)-D-glucans (approximately ~890 cm^−1^), associated with the β-configuration of glycosidic linkages within polysaccharides [28,31]. Considering that oats are widely recognized as a functional food due to their high content of soluble β-glucans and other bioactive compounds, the ATR–FTIR analysis performed in this study highlights spectral profiles characteristic of a polysaccharide-dominated matrix across all investigated samples. Under identical experimental conditions, sample BO exhibited the most pronounced spectral features associated with hydrophilic and polysaccharide components, observations that are consistent with reports in the literature describing oat varieties with a favorable nutritional and bioactive profile [26,27,28,29,30,31,32,33].

In the case of the analyzed samples, the cultivar OO presented the highest intensities of the bands at ~1150 cm^−1^ and ~1076 cm^−1^, followed by BO, while JO and SO recorded lower values (Table 4). Since β-glucans represent a major fraction of non-starch polysaccharides in oats, these differences in intensity suggest a relatively higher contribution of the β-glucan structure in OO and BO compared to JO and SO.

The band around ~997 cm^−1^ is associated with vibrations of the carbohydrate ring and may reflect changes in the arrangement of β-(1→3)/(1→4) bonds and the degree of branching of the glucan chains. The higher intensities observed in OO and BO in this region support differences in the structural organization of the β-glucan fraction between cultivars.

The band at ~860 cm^−1^, related to vibrations of β-glycosidic bonds, was present in all samples, but with maximum intensities in OO, which reinforces the hypothesis of a relatively more pronounced content of β-glucan structures in this cultivar.

Thus, based on the spectral profile and the relative intensities of the bands characteristic of polysaccharides, the following trend regarding the relative contribution of the β-glucan fraction can be suggested: OO > BO > JO ≈ SO.

It is important to note that FTIR provides a semi-quantitative and comparative assessment, not a direct quantification of β-glucans and does not aim to replace advanced structural analyses such as NMR or chromatographic techniques, since the bands may also include contributions from starch or other hemicelluloses. However, the systematic differences observed between cultivars indicate real variations in the structure and proportion of β-glucan polysaccharides.

### 2.5. Rheological Profile of Composite Flours Based on Black Oat (Avena strigosa) and White Oat (Avena sativa *L.*) Cultivars

The Mixolab is an advanced rheological measuring instrument that allows simultaneous evaluation of the behavior of proteins and starch in flours under controlled thermo-mechanical conditions. This tool is widely used to predict the technological quality of bread-making flours and composite mixtures intended for bread-making [34,35].

#### 2.5.1. Mixolab Profiler Index

**Figure 5:**
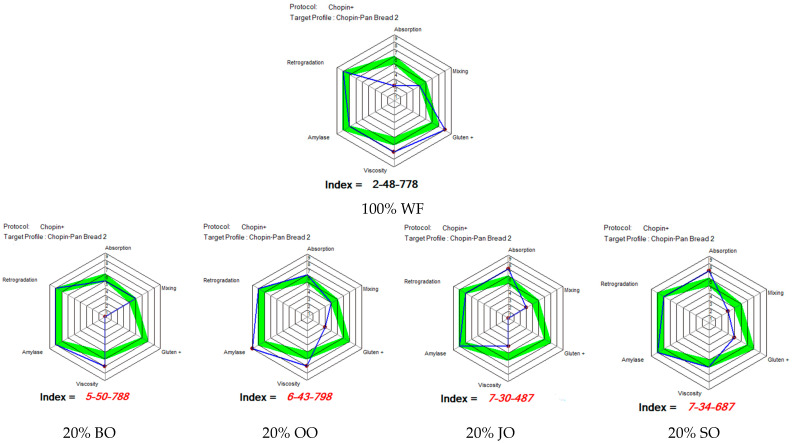
Mixolab profiler index for composite flours (100% WF, 20% BO, 20% OO, 20% JO, and 20% SO: blue line) compared with standard MIXOLAB profile (green line) for pan bread. 100% WF: 100% white wheat flour; 20% BO: 80% white wheat flour and 20% black oat flours, 20% OO: 80% white wheat flour and 20% Ovidiu cultivar oats flours, 20% JO: 80% white wheat flour and 20% Jeremy cultivar oat flours, 20% SO: 80% white wheat flour and 20% Sorin cultivar oat flours.

Figure 5 shows the Mixolab Profiler index, which expresses six technological parameters in the form of standardized indices (0–9). These indices facilitate comparison with recommended reference values for obtaining standard quality bread.

As part of this study, the criteria for evaluating formulations based on wheat flour partially substituted with different oat flours were defined based on the ranges recommended by Chopin Technologies for good bread. These criteria, which relate to absorption (5–6), kneading stability (4–5), gluten strength (6–7), viscosity (5–6), amylase activity (7–8) and retrogradation (7–8), make it possible to measure the effectiveness of these different oat flours as partial substitutes for wheat flour in the formulations studied.

**Figure 6:**
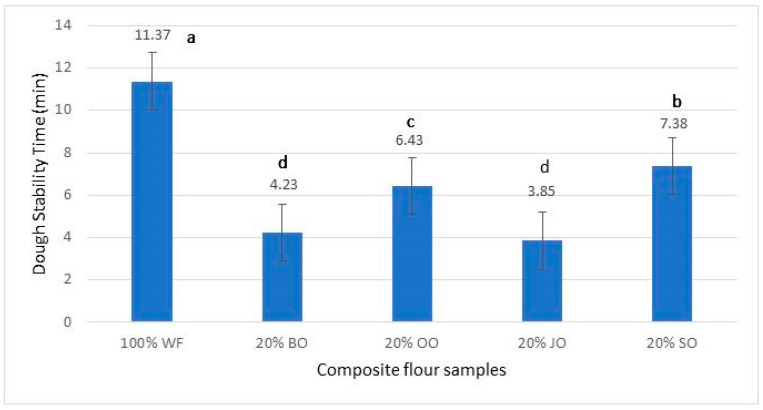
Dough stability of composite flour samples. BO: Black oat; OO: Ovidiu oat; JO: Jeremy oat; SO: Sorin oat, The values are expressed as mean values ± standard deviations of all measurements; data sharing different letters are significantly different (*p* < 0.05) according to Duncan test.

After analyzing Figure 6, it was established that the water absorption index of the sample with 100% WF is low (2), which is significantly below the optimal range. This behavior is typical of refined wheat flours, which are characterized by low dietary fiber content and limited water retention capacity [36]. On the other hand, kneading stability (index 4) and gluten strength (index 8) indicate a well-developed gluten network and good mechanical tolerance. The viscosity (7) and amylase activity (7) indices are within the recommended values for bread making, indicating adequate starch gelatinization and balanced enzyme activity [29]. The high retrogradation index (8) suggests optimal crumb stability during storage, associated with a slowdown in staling. Thus, the control flour has good technological performance, although its low water absorption may limit the yield and nutritional value of the bread.

The incorporation of 20% black oat flour (20% BO) led to a significant increase in water absorption (index 5), approaching the recommended range for bread making. This improvement is attributed to the high soluble fiber content of oats, particularly β-glucans, which have strong water-binding capacity [5]. However, the gluten strength index was zero, indicating major disruption of the gluten network. This alteration is attributable to the dilution of the gluten and the physical interference of the oat constituents. However, the high viscosity index (7) reflects the thickening effect of non-starch polysaccharides. The amylase activity index (8) and retrogradation index (8) are key markers for assessing fermentation quality and bread stability during storage [35]. These results suggest that black oats offer advantages in terms of hydration and nutrition, but that technological adjustments are needed to counteract the degradation of the protein network.

The formulation containing 20% oat flour from the Ovidiu cultivar demonstrated optimal absorption, with an index of 6, which is in line with the recommendations of the Mixolab Profiler index for Bread. However, the analysis revealed that gluten strength remained moderate, with an index of 3, suggesting a partial weakening of the gluten network. The high viscosity index (7) and very high amylase activity (index 9) suggest intense enzymatic activity. Such activity can promote fermentation, but also increase the risk of sticky crumb if not controlled. Nevertheless, the retrogradation index (8) suggests satisfactory stability of the bread during storage. Indeed, a comparative analysis of the Ovidiu cultivar with black oats reveals that, although the former has a more balanced technological profile, control of enzymatic activity remains necessary to ensure optimal quality and safety.

The incorporation of 20% Jeremy cultivar oat flour led to significantly higher water absorption (index 7), exceeding values considered optimal. This increase in water demand is likely due to the specific fiber composition and particle structure of the flour. Kneading stability (index 3) and gluten strength (index 0) reveal low mechanical tolerance and significant disruption of the gluten network. In addition, the reduced viscosity index (4) may be an indicator of an alteration in the starch –fiber matrix during the heating process [35]. Although retrogradation (index 7) is considered acceptable, analysis of the overall rheological profile indicates limited bread-making suitability for this cultivar at 20% substitution.

Among the formulations enriched with oats, the Sorin cultivar exhibited the most favorable technological behavior. Water absorption (index 7) and viscosity (index 6) were close to the recommended values, indicating balanced hydration and adequate starch gelatinization. It should be noted that gluten strength (index 4), although slightly below the optimal range, remained higher than that observed for the other cultivars. The amylase activity (8) and retrogradation (7) indices are key indicators of fermentation quality and crumb stability [35]. These results suggest that the Sorin cultivar interacts more favorably with wheat flour, likely due to differences in protein composition and the nature of β-glucans.

Analysis of formulations containing 20% oat flour revealed an increase in water absorption and viscosity, confirming the positive impact of oat fiber on dough hydration and structure. However, a decrease in gluten strength was observed, which is consistent with previous work on wheat-oat systems [37,38,39]. An in-depth analysis of the data collected by the Mixolab Profiler for Bread highlighted that the formulation containing 20% oat flour from the Sorin cultivar offers the best compromise between technological performance and nutritional enrichment. Formulations based on black oats and the Ovidiu cultivar also show promising potential, subject to technological adjustments such as the addition of enzymes or protein reinforcements. In contrast, the Jeremy cultivar shows limited bread-making capacity at the substitution rate considered.

#### 2.5.2. Dough Stability Time (Min)

Dough stability time is an essential rheological parameter, reflecting the ability of the gluten network to withstand mechanical action during kneading, and is closely correlated with dough processability and final bread quality. The values obtained indicate clear differences between the control bread made from wheat flour (100% WF) and the variants with the addition of 20% oat flour (Figure 6).

The dough obtained from 100% wheat flour showed the highest stability (11.37 min), confirming the development of a continuous and resistant gluten network, specific to flours intended for bakery. This behavior is consistent with the specialized literature, which highlights the role of gluten proteins (gliadin and glutenin) in maintaining dough stability during kneading [40,41].

The introduction of 20% oat flour resulted in an overall reduction in dough stability, an effect attributed to the dilution of gluten and the presence of non-gluten proteins, fibers, and oat-specific β-glucans. The lowest stability was observed for the 20% oat Jeremy bread (3.85 min), followed by 20% black oat (4.23 min). These values indicate a more fragile dough structure and reduced kneading tolerance, a phenomenon frequently reported in bakery formulations enriched with oats or other functional gluten-free cereals [42].

The 20% oat Ovidiu bread showed intermediate stability (6.43 min), suggesting a better balance between the residual gluten network and the structural components introduced by the oats. The highest stability among the oat variants was recorded for 20% Sorin oat (7.38 min), indicating superior rheological behavior compared to the other enriched formulations. This varietal difference can be explained by particularities of the chemical composition of Sorin oat, including favorable interactions between proteins, lipids and mineral fractions, which may contribute to the stabilization of the dough’s colloidal matrix.

In the recent literature, it has been demonstrated that oat lipids can exert a plasticizing effect on dough, while β-glucans increase viscosity and water retention capacity, but can reduce kneading tolerance when present in high amounts [43]. Therefore, the differences observed between the analyzed oat varieties suggest that not only the level of substitution, but also the genetic origin of the oat significantly influences dough stability.

Overall, the results indicate that, although the addition of oats negatively affects dough stability compared to wheat flour, certain varieties, such as Sorin, can maintain stability values compatible with the technological requirements of bakery production, while others require process adjustments (reduced kneading time, optimized hydration).

#### 2.5.3. Water Absorption (%)

**Figure 7:**
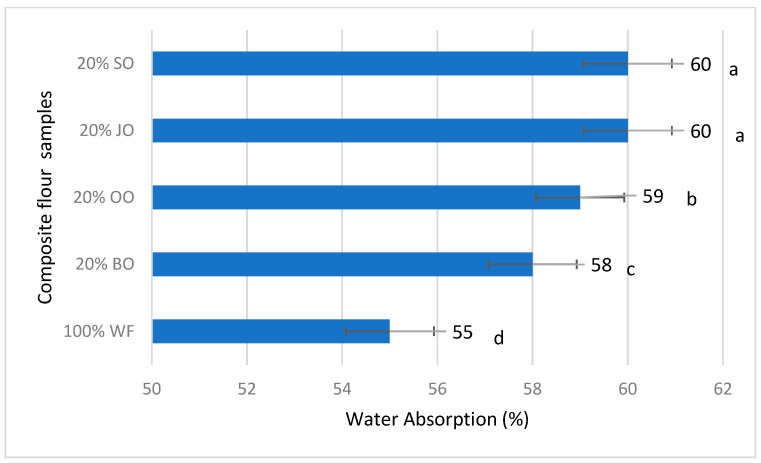
Water absorption of composite flour samples. BO: Black oat; OO: Ovidiu oat; JO: Jeremy oat; SO: Sorin oat, The values are expressed as mean values ± standard deviations of all measurements; data sharing different letters are significantly different (*p* < 0.05) according to Duncan test.

Water absorption capacity is a fundamental rheological parameter, reflecting the interaction between water and flour components (proteins, carbohydrates, fibers, and minerals), having a direct impact on the dough consistency and the baking process. The values obtained (Figure 7) indicate a clear increase in water absorption for all variants with the addition of 20% oat flour, compared to the control bread made from wheat flour (100% WF).

The dough obtained from 100% wheat flour presented the lowest water absorption (55%), a typical value for refined white flours, characterized by a low fiber and mineral content. In contrast, all variants enriched with oats required an additional water intake, the absorption increasing up to 58–60%, confirming the significant influence of oats on the rheological behavior of the dough.

The highest water absorption (60%) was observed for breads with 20% Sorin oat (20% SO) and 20% Jeremy oat (20% JO), followed by 20% Ovidiu oat (59%) and 20% black oat (58%). This hierarchy reflects the differences in chemical composition between the types of oats used and is consistent with the determined nutritional values. Thus, the variants with maximum absorption (20% JO and 20% SO) presented a higher content of proteins and lipids and a lower content of available carbohydrates, components known for their ability to bind water.

The increase in water absorption can be attributed mainly to the soluble fibers in oats, especially β-glucans, which form hydrophilic structures capable of retaining large amounts of water. Additionally, non-gluten proteins and the mineral fraction contribute to the hydration of the dough through electrostatic interactions and hydrogen bonds. In the case of black oats and the Sorin variety, the high content of mineral substances may explain, at least in part, the higher absorption compared to the control, even if the values do not reach the maximum level observed for Jeremy.

From a technological point of view, the increase in water absorption is an advantage for obtaining a wetter and more elastic core, but requires adjustments to the recipe and process (higher hydration, adapted kneading time). At the same time, this characteristic is closely related to the reduction in dough stability observed previously, suggesting that increased hydration favors viscosity, but can accelerate the weakening of the gluten network when oats are present in significant proportions.

Overall, the results confirm that the type of oat differentially influences the water absorption capacity, even at the same level of substitution, and this varietal variability must be taken into account in the formulation and optimization of bakery products enriched with oats.

### 2.6. Bread Quality Assessment

#### 2.6.1. Nutritional Profile

Partial replacement of wheat flour with 20% oat flour, regardless of the type of oat used, led to significant changes in the nutritional profile of the bread compared to the control obtained exclusively from wheat flour (100% WF) (Table 5). All variants enriched with oats presented a higher protein content, values ranging between 11.99% (WBSO) and 12.43% (WBJO), compared to 11.82% in the case of bread made from wheat flour. This increase can be attributed to the protein content of oats, known for its higher content of globular proteins and for a more balanced distribution of essential amino acids compared to wheat, although without functional gluten [40,41].

The lipid content also increased in all oat variants, reaching a maximum in the bread with 20% Jeremy oat (1.23%), compared to only 0.74% in the control. This trend reflects the specific composition of oat, which is characterized by a higher lipid content than other cereals, and is rich in unsaturated fatty acids, contributing beneficial effects on cardiovascular health. The differences observed between varieties suggest the existence of a genotypic effect on the accumulation of the lipid fraction in the finished product.

A notable increase was also observed for total mineral substances (ash). Bread with 20% black oat (WBBO) and that with 20% Sorin oat (WBSO) recorded the highest values (0.93%), almost three times higher than the control bread (0.32%). This result highlights the potential of oat, especially black oat and the Sorin variety, to improve the mineral density of bakery products, an aspect of major nutritional interest. The literature indicates that oats are an important source of K, Mg, Fe, Zn, and Mn, essential elements involved in energy metabolism and enzymatic function [42].

In terms of carbohydrates, the oat variants presented comparable or slightly reduced values compared to the wheat flour bread, the lowest value being recorded for the 20% oat Jeremy bread (41.92%). This reduction is explained by the dilution of the wheat starch fraction and the increase in the proportion of oat-specific dietary fibers, especially β-glucans, which are not fully reflected in the available carbohydrate fraction [43]. The reduction in digestible carbohydrates, correlated with the increased fiber intake, is associated with a potential lower glycemic index, an aspect frequently reported for oat-enriched bakery products [43].

From an energetic point of view, the nutritional value (kcal) of the bread was slightly higher for the variants with 20% black oat and Ovidiu oat (241.36 and 238.61 kcal), due to the higher lipid content, while the breads with 20% Jeremy and Sorin oat presented lower energy values (228.42 and 226.18 kcal), comparable or even lower than the control. These results suggest that the choice of the type of oats allows the adjustment of the energy density of the finished product depending on the nutritional objective pursued.

Overall, the addition of 20% oat flour significantly improved the protein, mineral, and lipid profile of the bread, accompanied by a moderate reduction in available carbohydrates, confirming the potential of oats as a functional ingredient in bakery products. The differences observed between black oat and cultivated varieties (Ovidiu, Jeremy, and Sorin) highlight the importance of the genetic factor in determining the nutritional quality of bakery products and support the need for comparative evaluation of local varieties for the development of value-added products.

Based on the nutritional data, the oat cultivar Jeremy emerged as the most suitable variety for breadmaking at a 20% substitution level, due to its superior protein and lipid contents, reduced carbohydrate fraction, and balanced energy value. While the bread with addition of Jeremy oat proved to be the most suitable in terms of protein and lipid enrichment, the bread fortified with Sorin cultivar showed superior mineral content, low lipidic and carbohydrate fractions and the lowest energy value, being recommended in a dietary diet.

Bread with the addition of 20% black oat recorded the highest content of total mineral substances, almost three times higher compared to the wheat flour control, highlighting a superior mineral density. This characteristic is particularly relevant from a nutritional point of view, considering the role of macro- and microelements in energy metabolism, enzymatic function, and bone health. The specialized literature indicates that black oat is a valuable source of K, Mg, Fe, Zn and Mn, essential elements that can contribute to reducing the risk of mineral deficiencies in the diet.

In addition, black oat is recognized for its high content of bioactive compounds, including polyphenols and pigments with antioxidant activity, which can confer additional functional properties to the finished products. The presence of these compounds, combined with the high level of minerals, supports the potential of black oat for the development of value-added bakery products, oriented towards consumer segments interested in functional and healthy foods.

From a technological perspective, although black oat has not reached the maximum values in terms of protein or lipid content, their distinct nutritional profile and significant contribution to the mineral enrichment of bread recommend them as an important complementary ingredient. Thus, the use of black oat in bakery products can be justified not only in terms of nutritional value, but also as a strategy for diversifying raw materials and capitalizing on less exploited genetic resources.

These results indicate that the optimal oat genotype depends on whether nutritional or functional criteria are prioritized. The integration of different types of oats in bakery recipes can contribute to the development of differentiated products, adapted to current market requirements and oriented towards promoting a healthy diet.

#### 2.6.2. Physical Characteristics of Different Types of Bread

Table 6 shows the quality criteria for different types of bread obtained. These are porosity (a key parameter of bread quality, directly linked to the dough’s ability to retain the gases produced during fermentation and to the stability of the protein network during baking), elasticity (an indicator of the crumb’s ability to regain its shape after compression, which is highly dependent on the integrity of the protein network and the distribution of air pockets), and the H/D ratio (a macroscopic parameter reflecting the bread’s ability to expand during baking).

An examination of the data presented in the above table shows that the incorporation of 20% oat flour into the bread composition (WBBO, WBOO, WBJO, and WBSO) resulted in significant changes in the crumb structure and physical properties compared to the control bread made from white wheat flour (WB). The highest porosity observed for WB (68.25%) reflects a well-developed gluten network, while the composite breads showed reduced porosity (54.44–59.69%), reflecting a lower gas retention capacity due to gluten dilution and interference from oat components (insoluble fiber, β-glucans) [44,45]. This reduction was accompanied by a decrease in elasticity, which was particularly marked for bread containing the Jeremy cultivar oats (WBJO), suggesting a more compact and less resilient crumb. These differences suggest that the impact of oats on elasticity is cultivar-dependent; i.e., it varies depending on the oat variety. Higher elasticity was correlated with a more beneficial interaction between the components of oats and the starch-gluten matrix, as well as a more homogeneous distribution of pores in the crumb [46].

Regarding the H/D ratio, the addition of oat flour led to a reduction in this ratio for all composite breads, with the lowest values recorded for WBJO (0.59 ± 0.05) and WBOO (0.61 ± 0.02). These results confirm the hypothesis that partial substitution of wheat flour with oats limits bread expansion. This limitation is due to the reduction in gluten network strength and the increase in dough viscosity. However, the analyses revealed that WBSO (0.62 ± 0.02) and WBBO (0.70 ± 0.01) bread had a higher relative expansion capacity, suggesting that certain oat cultivars are better able to preserve the rising properties of bread.

A joint analysis of porosity, elasticity, and the H/D ratio reveals significant variability between different oat cultivars. WBJO bread is characterized by limited technological performance, manifested by a more compact, less elastic crumb and reduced expansion. In contrast, WBBO and WBSO breads offer a more advantageous compromise between crumb structure and mechanical properties. These differences are attributable to the specific composition of oat cultivars, particularly their β-glucan content, flour particle size distribution, and interactions with wheat proteins. Previous research has established that β-glucans are likely to induce an increase in water retention while contributing to a stiffening of the dough structure. This dynamic has repercussions on the physical properties of bread, particularly its volume and texture [47,48].

### 2.7. Color Parameters

The color parameters of oat varieties, composite flours, and bread samples measured using the colorimeter method according to the CIE Lab* color system are presented in Table 7.

The instrumental color parameters highlight clear differences between the analyzed oat varieties. The brightness (L*) showed a progressive increase from the reference sample (67.85 ± 3.44) to the oat varieties, the highest value being recorded for the Sorin variety (78.47 ± 0.10). This indicates a lighter appearance of the flour/grains from this variety, the differences in brightness being associated in the specialized literature with variations in the composition of the peripheral layer of the grain, the fiber content and the degree of natural pigmentation of the different oat genotypes [49,50].

The a* coordinate (green–red) was the lowest in the reference sample (0.68 ± 0.60) and increased significantly in the oat varieties, reaching a maximum in Sorin (3.28 ± 0.50). Higher positive values indicate a tendency towards more reddish shades, a phenomenon also reported in other studies for colored oats or for varieties with higher content of phenolic compounds located in the outer layers of the grain.

Similarly, the b* coordinate (blue–yellow) showed a consistent increase from 8.50 ± 7.57 to 15.07 ± 0.00, indicating an intensification of yellow tones in the case of oat varieties, especially the Sorin variety. The literature shows that these differences can be correlated with the presence of natural pigments (of the carotenoid type) and with the composition of bran fractions, which vary between genotypes.

Total color difference (ΔE) relative to the wheat control ranged from 3.89 to 12.76, indicating that varietal differences in oats produced visible to pronounced changes in color, with the Sorin variety showing the greatest chromatic deviation.

Overall, the results confirm that the oat variety significantly influences the chromatic characteristics, with differences in L*, a* and b* reflecting genetic and compositional particularities of the grains. Such inter-cultivar variations have been previously reported and are considered indicators of biochemical and structural diversity among cultivars [49,50].

Similar trends are also observed in the case of composite flours with minimum value for L* parameter recorded for composite flour with 20% black oat (74.40 ± 5.55) and without significant differences between white oat varieties regarding this index.

The a* coordinate (green–red) and b* coordinate (blue–yellow) recorded maximum values in the case of Sorin composite flour, indicating an intensification of these colors for flours obtained with addition of this white oat variety. The addition of oats significantly influenced the bread color. Compared to wheat bread (WB), all oat samples showed lower L* values and higher a* and b* values, indicating a darker core and warmer (reddish–yellowish) hues. This behavior is frequently reported in breads made from composite flours and is associated with the presence of bran fractions, phenolic compounds and the intensification of Maillard reactions during baking.

The total color difference (ΔE) for bread samples with oat flour addition compared to the control was very high (14.96–21.51), confirming that the addition of oats produces visible and technologically relevant chromatic changes. The most pronounced differences were observed for WBBO and WBOO, indicating a greater contribution of these varieties to darkening and intensifying the core color. In food colorimetry, ΔE > 3 is already a difference visible to the naked eye, and values > 10 indicate a very obvious difference. This means that all oat breads are clearly chromatically different from the control [51,52].

Although the Sorin variety exhibited the largest color deviation in the oat flour samples, this trend was not maintained in bread, where color development is mainly governed by Maillard reactions, starch–protein interactions, and the dilution effect of wheat flour, rather than the intrinsic pigment composition of the oat variety.

## 3. Materials and Method

### 3.1. Raw Material and Sample Preparation

Black oat and conventional oat cultivars (Ovidiu, Jeremy, Sorin) were grown in the 2024 season under standard agronomic practices in the Banat region (Romania). The three white oat cultivars (Ovidiu, Jeremy, and Sorin) and the black oat variety included in this study were selected because they are widely grown and agronomically adapted cultivars, representing the types of oats that are realistically available for use in cereal processing. Therefore, the study was designed as an applied technological assessment, focusing on how representative cultivated varieties behave in wheat–oat composite bread systems. These varieties are widely cultivated under local pedoclimatic conditions and form the basis of regional oat production. Evaluating their technological performance and functional potential in wheat–oat composite bread systems is therefore of both scientific and practical significance, as existing research has rarely considered such locally relevant cultivars in a comprehensive technological and functional context.

After harvest, grains were cleaned, air-dried to a moisture content of approximately 12%, and stored in sealed polyethylene bags at 4 °C until analysis.

Prior to chemical analyses, grains were milled using a laboratory hammer mill (mesh size 0.5 mm). This particle size and the wholemeal nature of the flour contributed to the presence of coarse particles, which influenced the texture and surface appearance of the bread. The resulting wholemeal flours were used for the determination of proximate composition (protein, lipid, ash) and mineral profile (macro- and microelements). All analyses were performed in triplicate.

### 3.2. Determination of Humidity

Moisture content of flours and bread samples was determined according to the AOAC air-oven drying method and calculated as weight loss during drying, then expressed as a percentage of the initial sample weight [53].

### 3.3. Determination of Crude Protein

Crude protein content was determined by the Kjeldahl method according to AOAC method 979.09 [54]. Total nitrogen content (*n*, %) was calculated from the volume of titrant and converted to crude protein using a conversion factor of 6.25. Results were expressed as g protein per 100 g dry matter (DM).

### 3.4. Determination of Crude Lipid

Crude lipid content was determined by Soxhlet extraction according to AOAC 920.39 [53] using petroleum ether as solvent. Results were expressed as g per 100 g DM.

### 3.5. Determination of Total Mineral Substances (Ash)

Ash content was determined gravimetrically according to AOAC 923.03 by incineration at 550 °C [53]. Results were expressed as g per 100 g DM.

### 3.6. Determination of Carbohydrates

Carbohydrate content was calculated by difference, based on proximate composition, according to the approach commonly used in AOAC analyses for reporting “total available carbohydrates” [53].

### 3.7. Determination of Macro- and Microelements by Atomic Absorption Spectrometry

Macro- (K, Na, Ca, Mg) and microelements (Fe, Zn, Mn, Cu) were quantified by flame atomic absorption spectrometry (FAAS) using element-specific hollow cathode lamps. External calibration curves were prepared with certified standard solutions (R^2^ > 0.999). Element concentrations were expressed as mg/kg DM [54].

### 3.8. Determination of Total Polyphenol Content and Antioxidant Activity (DPPH Assay)

Total polyphenol content was determined using the Folin–Ciocalteu method, while antioxidant activity was evaluated by the DPPH radical scavenging assay [55].

First, an ethanolic extract was obtained from 1 g of sample (wheat or oat cultivars) with 10 mL of ethanol 70%. The extract was combined with diluted Folin–Ciocalteu reagent (1:10, *v*/*v*) and 1 mL of sodium carbonate solution (60 g/L). The reaction mixture was incubated at 50 °C for 30 min. Subsequently, the absorbance was recorded at 750 nm using a UV–Vis spectrophotometer (Specord 205; Analytik Jena AG, Jena, Germany). The results were expressed as milligrams of gallic acid equivalents (GAE) per 100 g of sample.

For DPPH assay an aliquot of 0.5 mL from the ethanolic extract prepared as above was mixed with 0.5 mL of a 0.004% (*w*/*v*) DPPH solution. The reaction mixtures were vortexed and incubated for 30 min at room temperature in the dark, and the absorbance was measured at 517 nm using a spectrophotometer (Specord 205; Analytik Jena AG, Jena, Germany). Ascorbic acid was used as a positive control, while the DPPH solution without extract served as the negative control. All measurements were performed in triplicate. The DPPH radical scavenging activity (*RSA*) was calculated according to the following equation:

*RSA* (%) = (1 − *A*/*A*_0_) × 100 (1)

where

- *A*_0_ represents the absorbance of the negative control
- *A* represents the absorbance of the sample extract

### 3.9. FTIR Spectra

Fourier transform infrared (FTIR) spectroscopy was performed using a Nicolet iS50 FT-IR spectrometer (Thermo Fisher Scientific, Waltham, MA, USA) equipped with an attenuated total reflectance (ATR) crystal. Infrared spectra were recorded in the spectral range of 4000–400 cm^−1^, with a resolution of 4 cm^−1^ and accumulation of 32 scans per sample. Prior to analysis, samples were pre-dried at 40 °C overnight to remove residual moisture that could interfere with spectral acquisition and interpretation. After drying, the samples were placed directly onto the ATR crystal, and spectra were collected under controlled conditions [54]. FTIR spectra were recorded for each oat flour sample in triplicate, with measurements performed at different points of the sample to minimize heterogeneity effects. The presented spectra represent averaged profiles from repeated acquisitions.

### 3.10. Preparation of Composite Flours Based on Different Oat Cultivars

Composite flours were prepared by substituting wheat flour (WF) with 20% oat flour (OF) (*w*/*w*), resulting in blends containing 80% wheat flour and 20% oat flour. A substitution level of 20% oat flour was selected as a technologically relevant compromise, providing functional enrichment while maintaining acceptable dough rheology and bread quality, as higher levels are known to impair gluten network development and loaf structure. This level was chosen to ensure sufficient gluten content for dough development while allowing a meaningful contribution of oat-derived nutrients and bioactive compounds. Substitution levels in the range of 15–25% have been widely reported as technologically suitable for wheat–oat composite breads, as higher levels tend to weaken the gluten network and adversely affect loaf volume and crumb structure [17,55]. The selected substitution level also reflects industrial relevance, as composite flours containing up to 20% oat flour are commonly used in commercial bakery formulations to improve dietary fiber, mineral content, and antioxidant capacity without compromising product quality [17,55]. The composite flours were subsequently used for MIXOLAB analysis and breadmaking experiments. Five composite flours and the corresponding 5 composite breads were prepared and analyzed, as presented in Table 8.

### 3.11. Dough Rheological Properties (Mixolab Assay)

The rheological behavior of wheat dough supplemented with black and white oat flour was evaluated using a Mixolab apparatus (Chopin Technologies, Paris, France), according to the ICC Standard No. 173 [56]. Wheat flour was partially substituted with 20% oat flour (*w*/*w*).

The standard Chopin + protocol was applied to assess protein behavior during mixing and heating, starch gelatinization, enzymatic activity, and starch retrogradation. The main Mixolab parameters recorded were: water absorption (%), dough development time (min), stability (min), C1 (initial dough consistency), C2 (protein weakening), C3 (starch gelatinization), C4 (hot gel stability), and C5 (starch retrogradation) [51].

All measurements were performed in duplicate, and values were expressed as mean ± SD.

### 3.12. Breadmaking Procedure

In order to evaluate the technological properties and the possibility of use in baking, composite flours based on white wheat flour and different oat cultivar flours were used in the preparation of bread samples, coded as presented in Table 2.

**Figure 8:**
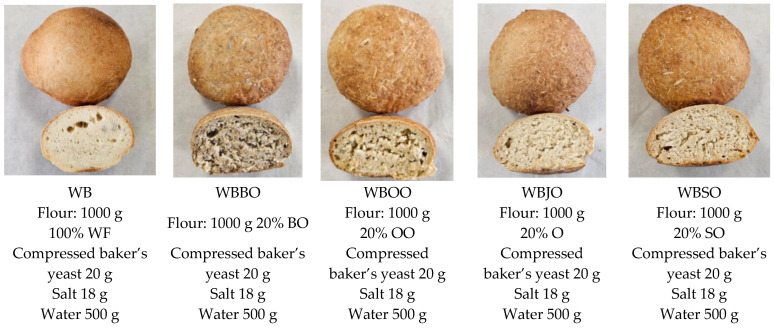
The bread samples (WB—bread with 100% white wheat flour—control sample, WBBO—bread with 80% white wheat flour and 20% black oat flour, WBOO—bread with 80% white wheat flour and 20% Ovidiu cultivar oat flour, WBJO—bread with 80% white wheat flour and 20% Jeremy cultivar oat flour, WBSO—bread with 80% white wheat flour and 20% Sorin cultivar oat flour).

Bread samples were prepared using a straight dough method [51]. The basic formulation consisted of wheat flour (or wheat flour partially replaced with black/white oat flour, as presented in Table 2), compressed baker’s yeast (2%), salt (1.8%), and water according to Mixolab-determined water absorption [56]. Doughs were mixed for 10 min, fermented at 30 °C and 85% relative humidity for 40 min, molded, proofed for 20 min, and baked at 220 °C for 25 min. Breads were cooled at room temperature for 2 h prior to analysis [51]. The samples obtained and the recipes are presented in Figure 8.

### 3.13. Bread Quality Evaluation

#### 3.13.1. Crumb Elasticity

Crumb elasticity was evaluated 24 h after baking using a compression–recovery test. Cylindrical crumb samples (height 6 cm) were carefully excised from the central part of the loaf to avoid crust influence. Compression was applied using a manual laboratory screw press equipped with parallel flat plates, providing vertical and uniform loading of the sample. The force was applied gradually by manual screw displacement until visible deformation of the crumb structure occurred. The deformation was then maintained for 1 min, monitored using a digital stopwatch. Because the compression was manually operated, the crosshead speed was not instrumentally controlled; however, all tests were conducted by the same operator under consistent handling conditions to ensure comparability among samples. After removal of the load, the sample was allowed to recover without external force. The initial height of the crumb cylinder before compression (*A*, cm) and the recovered height after the recovery period (*B*, cm) were measured using a digital caliper with 0.01 cm precision. *Elasticity* was calculated as the ratio of recovered height after deformation to initial height and expressed as a percentage, according to the following formula [57].

*Elasticity* (%) = *B*/*A* × 100 (2)

where

- *A* is the height of the core cylinder prior to the application of pressure (cm),
- *B* is the height of the cylinder after it has been returned to its original state following the pressure application (cm).

#### 3.13.2. Porosity

Porosity was expressed as the percentage of total void area relative to the total crumb area, according to the standard SR 91:2007 [57] using the formula:

(3) $$Porosity\left(\%\right)=\frac{V-\frac{m}{\rho }}{V}\times \;100$$

- *V* is the volume of the crumb cylinder (cm^3^),
- *m* is the mass of sample (g),
- *ρ* is the density of the compact crumb (g/cm^3^).

#### 3.13.3. Height-to-Diameter Ratio (H/D)

Loaf height (H) and diameter (D) were measured using a digital caliper at three different points per loaf. The height-to-diameter ratio (H/D) was calculated as an indicator of loaf shape and volumetric stability [58].

### 3.14. Color Analysis

The color parameters of oat varieties, composite flours and bread samples were measured using a colorimeter (Konica Minolta, Tokyo, Japan) according to the CIE Lab* color system, which is widely used for objective color evaluation. In this system, L* represents lightness (0 = black, 100 = white), a* indicates the position on the green (−) to red (+) axis, and b* describes the blue (−) to yellow (+) axis [58]. All determinations were performed in triplicate.

(4) $$\Delta E=\sqrt{(L^{*}-L^{0}{)}^{2}+(a^{*}-a^{0}{)}^{2}+(b^{*}-b^{0}{)}^{2}}$$

where

- L* represents lightness of oat sample and L^0^ lightness control (WF)
- a* value of oat sample and a^0^ value of control (WF) sample on green (−) to red (+) axis
- b* value of oat sample and b^0^ value of control (WF) sample on blue (−) to yellow (+) axis

### 3.15. Statistical Analysis and Correlations

Results are presented as mean ± standard deviation (SD). All experimental data were subjected to a one-way analysis of variance (ANOVA) followed by Duncan’s multiple range test to identify statistically significant differences (*p* < 0.05). For macro- and microelements composition, in order to explore the relationships between variables, Pearson’s linear correlation analysis was performed. Where strong and statistically significant correlations were observed, linear regression models were further established to quantify these associations. Subsequently, the data set was analyzed using two multivariate statistical techniques: Principal Component Analysis (PCA) and Cluster Analysis. All statistical computations and graphical representations were performed using the R statistical computing environment (v4.3.3; R Core Team, 2023, Vienna, Austria).

## 4. Conclusions

This study provides a comprehensive evaluation of the nutritional, bioactive, structural, and technological characteristics of different oat genotypes incorporated at a 20% substitution level in wheat-based composite flours and bread formulations. Unlike previous studies, the present paper directly compares black oat with locally developed oat cultivars under identical experimental conditions, demonstrating distinct genotype-dependent mechanisms governing mineral accumulation, antioxidant activity, and technological performance. By focusing on oat varieties characteristic of the Romanian agricultural area, this study delivers regionally relevant technological and functional insights that support their effective utilization in composite bakery formulations.

Therefore, the contribution of this work is not limited to compositional comparison but provides a system-level understanding of how oat variety influences the technological performance and functional enhancement of bread. Further studies involving direct microstructural analysis are needed to complement the rheological and physical findings and to elucidate the underlying structural mechanisms.

Overall, the results confirm that all the oat variants investigated retain the fundamental nutritional attributes of oats—namely, their contribution as valuable sources of high-quality proteins and lipids—while simultaneously influencing dough rheology and bread quality in a genotype-dependent manner. FTIR analysis revealed differences between cultivars in the spectral regions characteristic of polysaccharides, suggesting variations in the contribution of the β-glucan fraction between the oat varieties studied.

Among the genotypes evaluated, the Sorin cultivar showed the most favorable overall profile, combining high mineral content, strong antioxidant activity, and acceptable dough stability at a 20% substitution level. Black oat flour represents a valuable alternative for the development of bakery products targeting enhanced mineral and antioxidant properties, albeit with the need for minor process adjustments such as optimized hydration and kneading conditions. These findings provide useful guidance for both breeders and the bakery industry in selecting appropriate oat genotypes to develop functional bread products aligned with consumer demand for healthier foods.

Future studies should aim to optimize wheat–oat composite formulations by adjusting substitution levels and processing conditions according to oat genotype. Particular attention should be given to black oat and the Sorin cultivar to better understand the bioavailability of minerals and polyphenols and to improve dough stability through targeted technological interventions.

## Figures and Tables

**Table 1:** The proximate composition of black oat (*Avena strigosa*) and White Oat (*Avena sativa* L.) cultivars.

Nutrient	Black Oat (BO)	Ovidiu Oat (OO)	Jeremy Oat (JO)	Sorin Oat (SO)
Humidity (%)	9.52 ± 0.13 ^c^	9.69 ± 0.15 ^b^	9.52 ± 0.03 ^c^	12.61 ± 0.25 ^a^
Proteins (%)	12.39 ± 0.64 ^b^	13.48 ± 0.44 ^a^	12.48 ± 0.43 ^b^	13.13 ± 0.11 ^a^
Lipids (%)	4.09 ± 0.50 ^b^	3.24 ± 0.07 ^c^	3.27 ± 0.75 ^c^	4.64 ± 0.48 ^a^
Total mineral substances (%)	2.47 ± 0.36 ^a^	1.52 ± 0.59 ^c^	1.63 ± 0.26 ^b^	2.26 ± 0.46 ^a^
Carbohydrates (g/100 g)	71.53 ± 0.25 ^b^	72.07 ± 0.21 ^b^	73.1 ± 0.13 ^a^	67.36 ± 0.28 ^c^

The values are expressed as mean values ± standard deviations of all measurements; data within the same line sharing different superscripts are significantly different (*p* < 0.05); according to Duncan test.

**Table 2:** Macro and microelements composition of black oat (*Avena strigosa*) and white oat (*Avena sativa* L.) cultivars.

Element (mg/kg)	Black Oat	White Oat Cultivars		
		Ovidiu (OO)	Jeremy (JO)	Sorin (SO)
calcium	424.14 ± 1.07 ^c^	234.39 ± 1.27 ^d^	472.69 ± 1.05 ^b^	511.60 ± 1.57 ^a^
magnesium	655.14 ± 1.06 ^a^	247.82 ± 1.98 ^d^	530.76 ± 2.78 ^b^	448.31 ± 3.24 ^c^
potassium	1902.59 ± 1.75 ^b^	655.37 ± 1.36 ^d^	1603.34 ± 2.28 ^c^	2091.83 ± 2.73 ^a^
sodium	29.24 ± 0.22 ^c^	35.14 ± 0.32 ^a^	31.18 ± 1.05 ^b^	34.84 ± 0.92 ^a^
iron	26.98 ± 0.45 ^a^	22.71 ± 1.021 ^b^	25.78 ± 2.55 ^a^	27.52 ± 0.72 ^a^
manganese	27.91 ± 0.92 ^a^	21.82 ± 1.70 ^b^	22.04 ± 2.12 ^b^	23.94 ± 1.69 ^b^
copper	3.74 ± 0.21 ^a^	2.74 ± 0.15 ^b^	3.03 ± 0.09 ^b^	3.62 ± 0.25 ^a^
nickel	2.74 ± 0.10 ^a^	1.60 ± 0.26 ^c^	2.09 ± 0.36 ^b^	1.45 ± 0.18 ^c^
zinc	17.40 ± 0.13 ^a^	13.21 ± 0.36 ^c^	13.31 ± 0.26 ^c^	16.19 ± 0.19 ^b^

The values are expressed as mean values ± standard deviations of all measurements; data within the same line sharing different superscripts are significantly different (*p* < 0.05) according to Duncan test.

**Table 3:** Total polyphenol content and antioxidant activity (DPPH assay).

Samples	Polyphenol (mg GAE/100 g)	Antioxidant (% DPPH)
BO	408.59 ± 2.08 ^a^	54.68 ± 0.04 ^b^
OO	260.09 ±0.67 ^c^	49.95 ± 0.13 ^c^
JO	314.32 ± 0.10 ^b^	48.22 ± 0.07 ^d^
SO	209.89 ± 0.33 ^d^	56.19 ± 0.07 ^a^

The values are expressed as mean values ± standard deviations of all measurements; data within the same column sharing different superscripts are significantly different (*p* < 0.05); according to Duncan test.

**Table 4:** Characteristic FTIR absorption bands and their relative intensities in the polysaccharide region of oat cultivars.

No.	BO		OO		JO		SO	
	Peak	Intensity	Peak	Intensity	Peak	Intensity	Peak	Intensity
1	860.76	77.81	860.30	80.94	860.85	72.66	860.29	72.85
2	931.09	73.13	-	-	930.03	67.23	-	-
3	997.61	37.45	996.95	49.60	996.49	27.62	997.08	31.07
4	1076.42	59.92	1076.34	66.51	1076.42	52.60	1076.18	50.65
5	1149.85	70.93	1149.51	75.88	1149.83	64.00	1150.11	63.03

**Table 5:** The nutritional profile of wheat bread fortified with 20% different oat flour cultivars.

Nutrient/Sample	WBBO	WBOO	WBJO	WBSO	WB
Moisture (%)	38.1 ± 1.03 ^c^	39.39 ± 2.01 ^b^	42.06 ± 1.03 ^a^	41.62 ± 1.16 ^a^	42.18 ± 2.09 ^a^
Proteins (%)	12.17 ± 0.1 ^b^	12.34 ± 0.11 ^a^	12.43 ± 0.07 ^a^	11.99 ± 0.05 ^c^	11.82 ± 0.02 ^c^
Lipids (%)	0.94 ± 0.02 ^c^	1.05 ± 0.01 ^b^	1.23 ± 0.03 ^a^	0.95 ± 0.01 ^c^	0.74 ± 0.02 ^d^
Total mineral substances (%)	0.93 ± 0.01 ^a^	0.63 ± 0.02 ^b^	0.65 ± 0.01 ^b^	0.93 ± 0.01 ^a^	0.32 ± 0.01 ^c^
Carbohydrates (%)	46.06 ± 1.03 ^a^	44.95 ± 1.69 ^b^	41.92 ± 0.88 ^d^	42.41 ± 1.12 ^d^	43.4 ± 1.39 ^c^
Energy value (kcal)	241.36 ± 3.36 ^a^	238.61 ± 4.82 ^b^	228.42 ± 1.99 ^c^	226.18 ± 1.97 ^e^	227.53 ± 1.40 ^d^

WBBO—bread with 20% black oat, WBOO—bread with 20% Ovidiu oats, WBJO—bread with 20% oat Jeremy, WBSO—bread with 20% Sorin oat, WB—white wheat bread (control). The values are expressed as mean values ± standard deviations of all measurements; data within the same line sharing different superscripts are significantly different (*p* < 0.05); according to Duncan test.

**Table 6:** Physical characteristics of different types of bread (WBBO, WBOO, WBJO, WBSO, and WB).

Samples	WBBO	WBOO	WBJO	WBSO	WB
Porosity (%)	59.69 ± 1.87 ^b^	56.54 ± 1.59 ^d^	54.44 ± 1.68 ^e^	57.7 ± 1.57 ^c^	68.25 ± 1.77 ^a^
Elasticity (%)	92.88 ± 1.70 ^a^	85.65 ± 1.72 ^b^	73.97 ± 1.88 ^c^	86.18 ± 1.16 ^b^	93.33 ± 2.11 ^a^
Height/Diameter ratio (H/D)	0.70 ± 0.01 ^b^	0.61 ± 0.02 ^c^	0.59 ± 0.05 ^d^	0.62 ± 0.02 ^c^	0.72 ± 0.04 ^a^

The values are expressed as mean values ± standard deviations of all measurements; data within the same line sharing different superscripts are significantly different (*p* < 0.05); according to Duncan test.

**Table 7:** Color parameters of oat samples, composite flours and bread samples.

Samples	L*	a*	b*	ΔE
Oat variety				
100% WF	67.85 ± 3.44 ^e^	0.68 ± 0.60 ^c^	8.50 ± 7.57 ^d^	-
100% BO	70.68 ± 0.10 ^d^	2.22 ± 0.05 ^b^	10.68 ± 0.00 ^c^	3.89 ^d^
100% OO	73.94 ± 4.45 ^c^	2.28 ± 0.08 ^b^	12.03 ± 1.81 ^b^	7.22 ^c^
100% JO	74.45 ± 3.29 ^b^	2.35 ± 0.13 ^b^	12.84 ± 1.91 ^b^	8.07 ^b^
100% SO	78.47 ± 1.00 ^a^	3.28 ± 0.50 ^a^	15.07 ± 1.00 ^a^	12.76 ^a^
Composite flours				
20% BO	74.40 ± 5.55 ^b^	0.71 ± 0.47 ^c^	8.53 ± 5.70 ^c^	-
20% OO	87.34 ± 0.01 ^a^	0.87 ± 0.00 ^b^	9.73 ± 0.01 ^b^	-
20% JO	87.77 ± 0.73 ^a^	0.82 ± 0.08 ^b^	9.81 ± 0.13 ^b^	-
20% SO	87.52 ± 0.01 ^a^	1.22 ± 0.01 ^a^	10.26 ± 0.00 ^a^	-
Breads				
WB	82.25 ± 1.36 ^a^	1.42 ± 0.45	12.34 ± 5.30 ^d^	-
WBBO	60.92 ± 0.01 ^d^	2.99 ± 0.01	14.66 ± 0.00 ^c^	21.51 ^a^
WBOO	62.17 ± 2.10 ^c^	2.86 ± 0.20	15.70 ± 1.71 ^b^	20.41 ^b^
WBJO	68.52 ± 0.09 ^b^	2.80 ± 0.01	18.12 ± 0.02 ^a^	14.96 ^c^
WBSO	68.26 ± 0.56 ^b^	3.11 ± 0.41	18.45 ± 0.56 ^a^	15.36 ^d^

The values are expressed as mean values ± standard deviations of all measurements; data within the same line sharing different superscripts are significantly different (*p* < 0.05); according to Duncan test.

**Table 8:** Composite flours and breads based on wheat and black and white oat cultivars.

Composite Flours Code	Composite Breads Code	Flours Composition
100% WF	WB	100% white wheat flour (control sample)
20% BO	WBBO	80% white wheat flour and 20% black oat flours
20% OO	WBOO	80% white wheat flour and 20% Ovidiu cultivar oat flours
20% JO	WBJO	80% white wheat flour and 20% Jeremy cultivar oat flours
20% SO	WBSO	80% white wheat flour and 20% Sorin cultivar oat flours

## Data Availability

The original contributions presented in the study are included in the article; further inquiries can be directed to the corresponding author.

## References

[1] Butt M.S., Tahir-Nadeem M., Khan M.K.I., Shabir R., Butt M.S. (2008). Oat: Unique among the cereals. Eur. J. Nutr..

[2] El Khoury D., Cuda C., Luhovyy B.L., Anderson G.H. (2012). Beta-glucan: Health benefits in obesity and metabolic syndrome. J. Nutr. Metab..

[3] Biel W., Bobko K., Maciorowski R. (2009). Chemical composition and nutritive value of husked and naked oats grain. J. Cereal Sci..

[4] Leszczyńska D., Wirkijowska A., Gasiński A., Średnicka-Tober D., Trafiałek J., Kazimierczak R. (2023). Oat and oat processed products—Technology, composition, nutritional value, and health. Appl. Sci..

[5] Alemayehu G.F., Forsido S.F., Tola Y.B., Amare E. (2023). Nutritional and phytochemical composition and associated health benefits of oat (*Avena sativa*) grains and oat-based fermented food products. Sci. World J..

[6] Sterna V., Zute S., Brunava L. (2016). Oat grain composition and its nutrition benefit. Agric. Agric. Sci. Procedia.

[7] McLauchlan J., Tyler A.I.I., Orfila C., Sarkar A. (2026). Characterisation of an oat protein–beta-glucan co-extract. Food Hydrocoll..

[8] Brindzová L., Čertík M., Rapta P., Zalibera M., Mikulajová A., Takácsová M. (2008). Antioxidant activity, β-glucan and lipid contents of oat varieties. Czech J. Food Sci..

[9] Čech M., Ivanišová E., Hozlár P., Tokár M., Zagula G., Gumul D., Kačániová M., Sterczyńska M., Haščík P. (2021). Nutritional composition, biological activity and technological properties of new Slovakian black oat varieties. J. Microbiol. Biotechnol. Food Sci..

[10] Ivanišová E., Čech M., Hozlár P., Zaguła G., Gumul D., Grygorieva O., Makowska A., Kowalczewski P.Ł. (2023). Nutritional, Antioxidant and Sensory Characteristics of Bread Enriched with Wholemeal Flour from Slovakian Black Oat Varieties. Appl. Sci..

[11] Varga M., Jójárt R., Fónad P., Mihály R., Palágyi A. (2018). Phenolic Composition and Antioxidant Activity of Colored Oats. Food Chem..

[12] Chmelová D., Ondrejovič M., Havrlentová M., Hozlár P. (2015). Antioxidant Activity in Naked and Hulled Oat (*Avena sativa* L.) Varieties. J. Microbiol. Biotechnol. Food Sci..

[13] Ibrahim M.S., Ahmad A., Sohail A., Asad M.J. (2020). Nutritional and Functional Characterization of Different Oat (*Avena sativa* L.) Cultivars. Int. J. Food Prop..

[14] Kim I.S., Hwang C.W., Yang W.S., Kim C.H. (2021). Multiple Antioxidative and Bioactive Molecules of Oats (*Avena sativa* L.) in Human Health. Antioxidants.

[15] Jakobsone I., Zute S., Bleidere M., Kantane I., Ece L., Bartkevics V. (2019). Macro and Trace Elements in Oat Cultivars Bred in Latvia. Zemdirb. Agric..

[16] Alemayehu G.F., Forsido S.F., Tola Y.B., Teshager M.A., Assegie A.A., Amare E. (2021). Proximate, Mineral and Anti-Nutrient Compositions of Oat Grains (*Avena sativa*) Cultivated in Ethiopia: Implications for Nutrition and Mineral Bioavailability. Heliyon.

[17] Dossa S., Rinovetz A., Neagu C., Stoin D., Lalescu D., Jianu C., Radulov I., Serpe L., Brinzeu A., Alexa E. (2026). Impact of Sunflower (*Helianthus annuus*) Seed Meal Use on the Nutritional, Phytochemical, Rheological, Physicochemical, and Sensory Quality of Wheat Bread. Appl. Sci..

[18] Kraisig A.R., da Silva J.A.G., Carvalho I.R., Colet C.D.F., Fachinetto J., da Rosa Fraga D., Conceição G.M., Peter C.L., da Rosa J.A., Basso N.C.F. (2023). Biofortification via Foliar Application of Zinc in Oat Grains and the Effects on Nutritional Quality and Productivity Indicators. Aust. J. Crop Sci..

[19] FeedTables Oats: Mineral Composition Tables (Zn, Cu, etc.). https://www.feedtables.com/content/oats.

[20] Mrázková M., Sumczynski D., Šenkárová L., Salek R.N. (2025). Dietary Intakes and Exposures to Minerals and Trace Elements from Cereal-Based Mixtures: Potential Health Benefits and Risks for Adults. Nutrients.

[21] Jordan-Meille L., Holland J.E., McGrath S.P., Glendining M.J., Thomas C.L., Haefele S.M. (2021). The grain mineral composition of barley, oat and wheat on soils with pH and soil phosphorus gradients. Eur. J. Agron..

[22] Sang S., Chu Y. (2017). Whole Grain Oats, More than Just a Fiber: Role of Unique Phytochemicals. Mol. Nutr. Food Res..

[23] Zieliński H., Kozłowska H. (2000). Antioxidant Activity and Total Phenolics in Selected Cereal Grains and Their Different Morphological Fractions. J. Agric. Food Chem..

[24] Gangopadhyay N., Hossain M.B., Rai D.K., Brunton N.P. (2015). A Review of Extraction and Analysis of Bioactives in Oat and Barley and Scope for Use of Novel Food Processing Technologies. Molecules.

[25] Kaur H., Gill B.S., Karwasra B.L. (2018). In vitro digestibility, pasting, and structural properties of starches from different cereals. Int. J. Food Prop..

[26] Zhu K., Aykas D.P., Anderson N., Ball C., Plans M., Rodriguez-Saona L. (2022). Nutritional quality screening of oat groats by vibrational spectroscopy using field-portable instruments. J. Cereal Sci..

[27] Deng G., Nagy C., Yu P. (2023). Combined molecular spectroscopic techniques (SR-FTIR, XRF, ATR-FTIR) to study physiochemical and nutrient profiles of Avena sativa grain and nutrition and structure interactive association properties. Crit. Rev. Food Sci. Nutr..

[28] Suraiya S., Jang W.J., Haq M., Kong I.S. (2024). Isolation and characterization of β-glucan containing polysaccharides from *Monascus* spp. using Saccharina japonica as submerged fermented substrate. Polysaccharides.

[29] Sujka K., Koczoń P., Ceglińska A., Reder M., Ciemniewska-Żytkiewicz H. (2017). The application of FT-IR spectroscopy for quality control of flours obtained from polish producers. J. Anal. Methods Chem..

[30] Christodoulou P., Athanasopoulou S., Ladika G., Konteles S.J., Cavouras D., Sinanoglou V.J., Kritsi E. (2025). Decoding Plant-Based Beverages: An Integrated Study Combining ATR-FTIR Spectroscopy and Microscopic Image Analysis with Chemometrics. App. Chem..

[31] Majumdar A., Gil-González A.B., Grau A.B., Sardari R.R., Larsson O., Thyagarajan A., Zambrano J.A. (2024). Macromolecular characterization of high β-glucan oat lines. Heliyon.

[32] Synytsya A., Novak M. (2014). Structural analysis of glucans. Annals Transl. Med..

[33] Paudel D., Dhungana B., Caffe M., Krishnan P. (2021). A review of health-beneficial properties of oats. Foods.

[34] Dubat A. (2010). A new AACC International approved method to measure rheological properties of a dough sample. Cereal Foods World.

[35] Chopin Applications Laboratory (2009). Mixolab Applications Handbook. Rheological and Enzymatic Analysis.

[36] Rosell C.M., Rojas J.A., De Barber C.B. (2001). Influence of hydrocolloids on dough rheology and bread quality. Food Hydrocoll..

[37] Hager A.-S., Ryan L.A.M., Schwab C., Gänzle M.G., O’dOherty J.V., Arendt E.K. (2011). Influence of the soluble fibres inulin and oat β-glucan on quality of dough and bread. Eur. Food Res. Technol..

[38] Mei Z., Wang W., Feng X., Liu M., Peng S., Chen L., Chen H., Lin S. (2024). Effect of soluble oat β-glucan and tea polyphenols on the rheological properties and microstructure of wheat dough. LWT.

[39] Ruja A., Cozma A., Cozma B., Horablaga N.M., Dinulescu C., Alexa E., Buzna C., Cocan I., Berbecea A., Dossa S. (2024). Nutritional, Phytochemical, and Rheological Profiles of Different Oat Varieties and Their Potential in the Flour Industry. Agronomy.

[40] Cappelli A., Cini E. (2021). Challenges and Opportunities in Wheat Flour, Pasta, Bread, and Bakery Product Production Chains: A Systematic Review of Innovations and Improvement Strategies to Increase Sustainability, Productivity, and Product Quality. Sustainability.

[41] Shewry P.R., Hey S.J. (2015). The Contribution of Wheat to Human Diet and Health. Food Energy Secur..

[42] Hu H., Lin H., Xiao L., Guo M., Yan X., Su X., Liu L., Sang S. (2022). Impact of Native Form Oat β-Glucan on the Physical and Starch Digestive Properties of Whole Oat Bread. Foods.

[43] Bieniek A., Buksa K. (2024). The Influence of Oat β-Glucans of Different Molar Mass on the Properties of Gluten-Free Bread. Molecules.

[44] Flander L., Suortti T., Katina K., Poutanen K. (2011). Effects of wheat sourdough process on the quality of mixed oat-wheat bread. LWT Food Sci. Technol..

[45] Mariotti M., Lucisano M., Ambrogina P.M. (2006). Development of a baking procedure for the production of oat-supplemented wheat bread. Int. J. Food Sci. Technol..

[46] Scanlon M.G., Zghal M.C. (2001). Bread properties and crumb structure. Food Res. Int..

[47] Hager A.-S., Arendt E.K. (2013). Influence of hydroxypropylmethylcellulose (HPMC), xanthan gum and their combination on loaf specific volume, crumb hardness and crumb grain characteristics of gluten-free breads based on rice, maize, teff and buckwheat. Food Hydrocoll..

[48] Lazaridou A., Biliaderis C.G. (2007). Molecular aspects of cereal β-glucan functionality: Physical properties, technological applications and physiological effects. J. Cereal Sci..

[49] Lee N.-Y., Ha K.-Y. (2011). Quality Characteristics of Bread Added with Oat Flours. Korean J. Crop Sci..

[50] He X., Li X., Chen D., Huang S., Tao N. (2023). Effect on bread properties of partial substitution of wheat flour with oat flour and flour from oat grain germinated in the light or dark. Int. J. Food Sci. Technol..

[51] Ramos-Souza C., Bandoni D.H., de Rosso V.V. (2025). Color Development in Carotenoid-Enriched Bigels: Effects of Extraction Method, Saponification, and Oleogel-to-Hydrogel Ratios on CIELAB Parameters. Gels.

[52] Rodríguez R., Murgui X., Rios Y., Puértolas E., Pérez I. (2025). Application of Microwaves to Reduce Checking in Low-Fat Biscuits: Impact on Sensory Characteristics and Energy Consumption. Foods.

[53] AOAC International (2016). Official Methods of Analysis of AOAC International.

[54] Dossa S., Neagu C., Lalescu D., Negrea M., Stoin D., Jianu C., Berbecea A., Cseh L., Rivis A., Suba M. (2025). Evaluation of the Nutritional, Rheological, Functional, and Sensory Properties of Cookies Enriched with Taro (*Colocasia esculenta*) Flour as a Partial Substitute for Wheat Flour. Foods.

[55] Fluerasu D., Neagu C., Dossa S., Negrea M., Jianu C., Berbecea A., Stoin D., Lalescu D., Brezovan D., Cseh L. (2025). The Use of Whey Powder to Improve Bread Quality: A Sustainable Solution for Utilizing Dairy By-Products. Foods.

[56] Whole Meal and Flour from T. aestivum–Determination of Rheological Behavior as a Function of Mixing and Temperature Increase.

[57] Association ARS (2007). Romanian Standard for Bread, Confectionery and Bakery Specialties—Methods of Analysis.

[58] https://www.konicaminolta.com/instruments/knowledge/color/pdf/color_communication.pdf.

